# Output of a valveless Liebau pump with biologically relevant vessel properties and compression frequencies

**DOI:** 10.1038/s41598-021-90820-4

**Published:** 2021-06-01

**Authors:** Rubina Davtyan, Narine A. Sarvazyan

**Affiliations:** Department of Pharmacology and Physiology, School of Medicine and Health Science, The George Washington University, 2300 Eye Street NW, Washington, DC 20037 USA

**Keywords:** Biophysics, Developmental biology, Physiology, Systems biology, Cardiology, Engineering

## Abstract

Liebau pump is a tubular, non-peristaltic, pulsatile pump capable of creating unidirectional flow in the absence of valves. It requires asymmetrical positioning of the pincher relative to the attachment sites of its elastic segment to the rest of the circuit. Biological feasibility of such valveless pumps remains a hotly debated topic. To test the feasibility of the Liebau-based pumping in vessels with biologically relevant properties we quantified the output of Liebau pumps with their  compliant segments made of a silicone rubber that mimicked the Young modulus of soft tissues. The lengths, the inner diameters, thicknesses of the tested compliant segments ranged from 1 to 5 cm, 3 to 8 mm and 0.3 to 1 mm, respectively. The compliant segment of the setup was compressed at 0.5–2.5 Hz frequencies using a 3.5-mm-wide rectangular piston. A nearest-neighbor tracking algorithm was used to track movements of 0.5-mm carbon particles within the system. The viscosity of the aqueous solution was varied by increased percentage of glycerin. Measurements yielded quantitative relationships between viscosity, frequency of compression and the net flowrate. The use of the Liebau principle of valveless pumping in conjunction with physiologically sized vessel and contraction frequencies yields flowrates comparable to peristaltic pumps of the same dimensions. We conclude that the data confirm physiological feasibility of Liebau-based pumping and warrant further testing of its mechanism using excised biological conduits or tissue engineered components. Such biomimetic pumps can serve as energy-efficient flow generators in microdevices or to study the function of embryonic heart during its normal development or in diseased states.

## Introduction

In the 1950s, the German cardiologist Gephardt Liebau came up with the concept and an experimental proof of a new valveless pumping mechanism that subsequently acquired his name^1,2^. The Liebau pump creates flow by repetitive compression of a small part of a compliant valveless tube connected to a pair of stiff tubing on both ends. It requires asymmetrical positioning of the pincher element relative to the junctions between the compliant tube and the stiff segments. The created flow is pulsative and the relationship between the mean flowrate and the compression frequency is nonlinear.

Liebau argued that such a mechanism of valveless pumping can be involved in human circulation. It was later proposed that it represents an early evolutionary mechanism to circulate blood and, as such, may be present in several species of invertebrates and early vertebrates that had poorly developed or no heart valves^3,4^. In 2006, in vivo imaging of blood cell movement in the hearts of zebrafish embryos provided the first direct evidence that a Liebau-like pumping mechanism may be present during the development^5^.

The pioneering work of Liebau from the mid-1950s was largely forgotten as he did not find many followers in the field of cardiology. Instead several generations of theoretical physicists and mathematicians tried to model this intriguing mechanism using a variety of numerical approaches, often yielding conflicting outcomes^6–13^. Therefore, it became important to test this phenomenon experimentally. As of today, several groups have published experimental data using different variations of the Liebau pumps^14–19^. However, with no exceptions, these studies used at least one key parameter that laid outside the biological scales of these putative pumps. Among such parameters were vessel dimensions, compression frequency, fluid viscosity, or the elastic modulus of the compliant segment of the pump. The goal of this study was to create and experimentally test a simple model of the Liebau pump that operates within the range of parameters likely to be encountered biologically.

## Methods

### Protocol to create compliant segments

**Figure 1:**
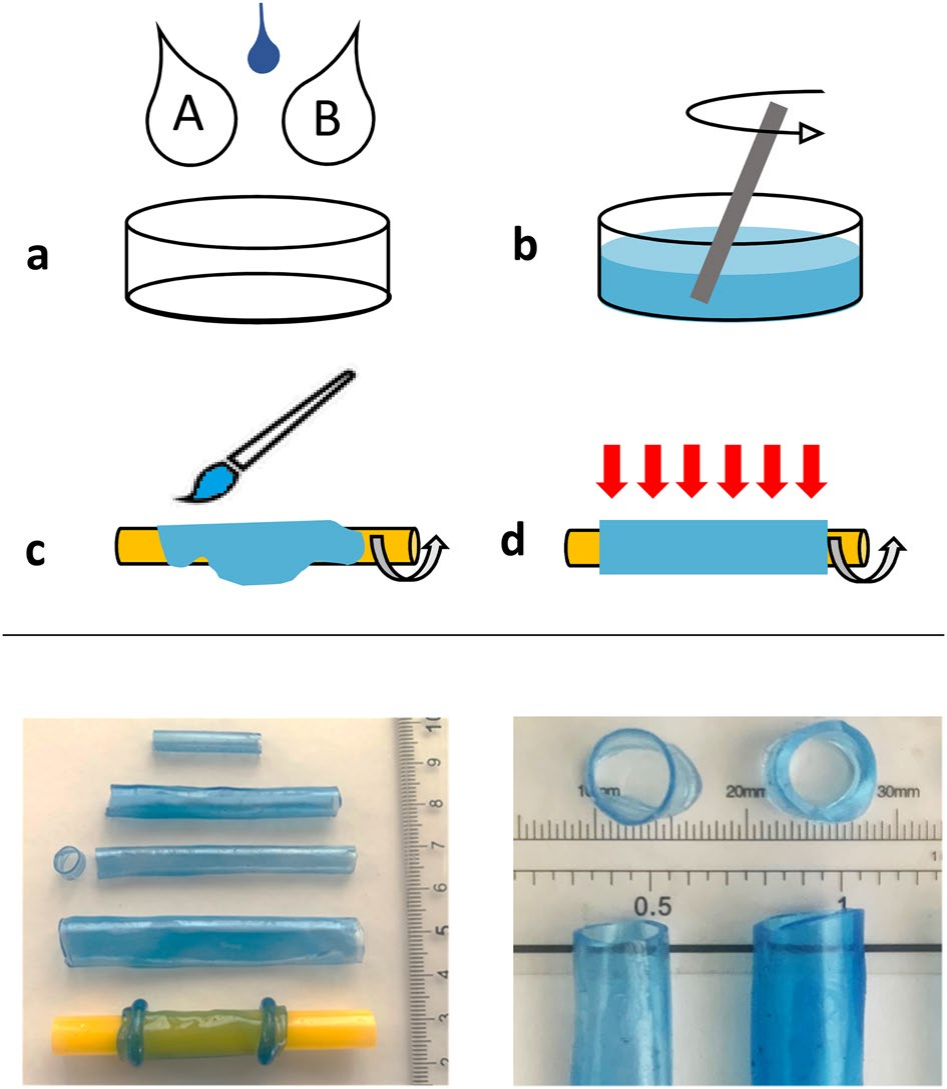
The creation of custom-sized highly compliant vessels. Top: Cartoons of the steps to create highly compliant tubes with the desired dimensions. (**a**) Components A and B of the Magikmold P-508 Silicone Rubber were mixed 1:1 by weight and a small volume of silicone dye was added; (**b**) careful mixing to avoid air bubbles was carried out; (**c**) the polymer mixture was then painted over a rotating mandrel; and (**d**) the stream of hot air helped to evenly distribute the mixture and promoted quick polymerization. Bottom: The image on the left shows a few samples of created tubes. The bottom tube is shown while being rolled off from the mandrel. The image on the right illustrates the evenness and wall thickness of the cross-sectioned tube samples.

A one-to-one mixture of Part A and B Magikmold P-508 Silicone Rubber was used to create an arrangement of compliant segments for the Liebau setup (Fig. 1). Weighted ingredients A and B were combined and carefully mixed to avoid air bubbles. A small amount of silicone dye was added to visualize the final product (~ 1–2 µl to 10 ml of mixture volume). Adding color to the polymer mixture ensured thorough mixing of the two components and helped to visualize its distribution over the rotating mandrel. It also enabled us to exclude conduits that had holes, trapped air bubbles or any visible heterogeneity in wall thickness. The colored silicone mixture was layered over cylindrical polypropylene mandrels of a chosen diameter, which were then rotated at a rate of 1–2 turns/s while being heated by a stream of hot air. The combination of heat and rotation led to an even distribution of the polymer across mandrel surface, while the silicone mixture was still in its liquid state. After ~ 10 min, the silicone mixture was no longer a liquid and did not require rotation to retain its shape. The conduits were then left at room temperature for another 24 h to complete polymerization, after which they were rolled off the mandrels. For the final tubes used in the experiments, the average standard deviation from their mean thickness value was found to be 10.3 ± 1.7%. The latter was calculated from color intensity of the image of the cross-sectioned and flattened tube surface, similarly to the methods we’ve described previously^20^.

### Young modulus determination

A rectangular strip of the above-described polymerized silicone rubber was connected to a set of weights and the degree of its stretch was measured. The Young modulus was determined as per formula E = longitudinal stress/longitudinal strain = g*m*L/A*ΔL, where g is the gravitational constant, m is the attached weight, A is the cross-sectional area and L is the length of the strip. The linear part of the stress/stretch curve extended up to 100% of the elongation values, therefore the Young modulus was equal to the 100% secant modulus. The measurements were repeated three times and yielded an average value of 47 ± 4 kPa.

### Experimental setup

**Figure 2:**
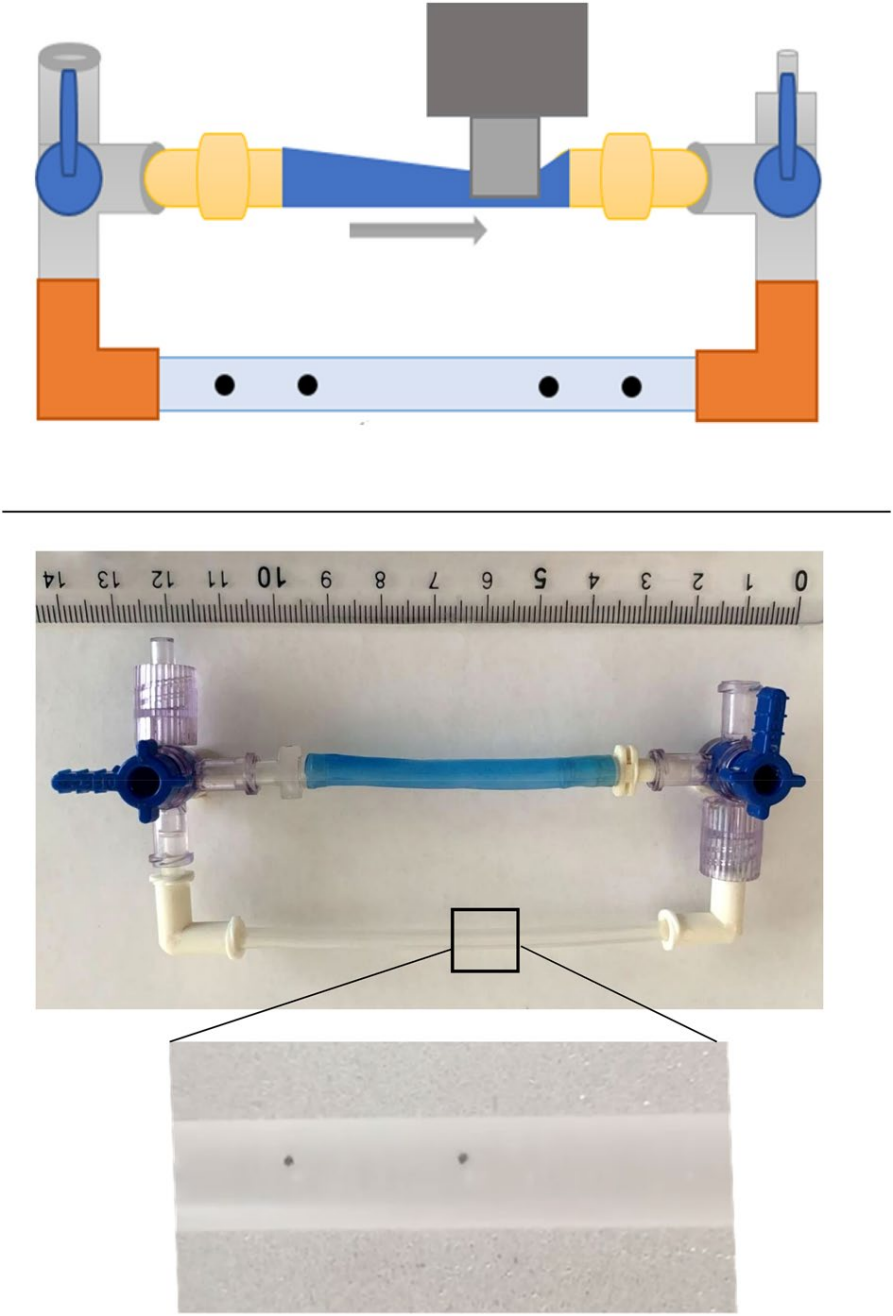
Experimental setup to test the Liebau pumping mechanism. Top: Cartoon of the experimental setup. The pincher (dark gray) is pressed onto the silicone rubber tube (blue) with specified frequency. The silicone rubber tube is connected to two 3-way stopcocks (light gray) via Teflon plastic connectors (yellow). Transparent Tygon tubing is connected to the stopcocks via two elbow connectors (orange). The fluid is injected through one of the stopcocks, while air bubbles eliminated through the other. The camera is positioned over the transparent part of the Tygon tubing for particle tracking. This cartoon pictures the pincher to be on the very edge of the silicone tube. Bottom: The actual image of the setup assembly with a closeup of the transparent section from which the images of the moving particles were taken.

The basic configuration consisted of a closed loop system, with inlet and outlet made from two polycarbonate three-way Luer stopcocks (Fig. 2). The stopcocks were used to fill the system with the fluid and remove the air bubbles. Two female-to-female nylon Luer elbow connectors linked the stopcocks to a piece of a transparent Tygon tubing of an adjustable length. The Tygon tubing had an outer diameter of 4 mm, an inner diameter of 3 mm and an estimated Young modulus of 11 MPa. Tight connection of the silicone segment to the three-way stopcocks was ensured by matching its diameter to the diameter of the plastic connectors and strengthening it using surgical thread. Black carbon particles sized ~ 0.5 mm were added to the fluid to track the velocity of the flow. The pincher was 3.5 mm wide, had a rectangular shape and was positioned to fully compress the lumen. It was driven by an actuator which was connected to a cyclic time relay Andeli DH48s-s with a time resolution of 0.1 s. The range of tested frequencies was 0.5–2.5 Hz, with the compression time held constant at 0.2 s, while the time off was changed to 0.2, 0.3,0.5, 0.8 and 1.8 s, yielding 2.5, 2, 1.5, 1, and 0.5 Hz compression rates, respectively.

### Flowrate measurements

**Figure 3:**
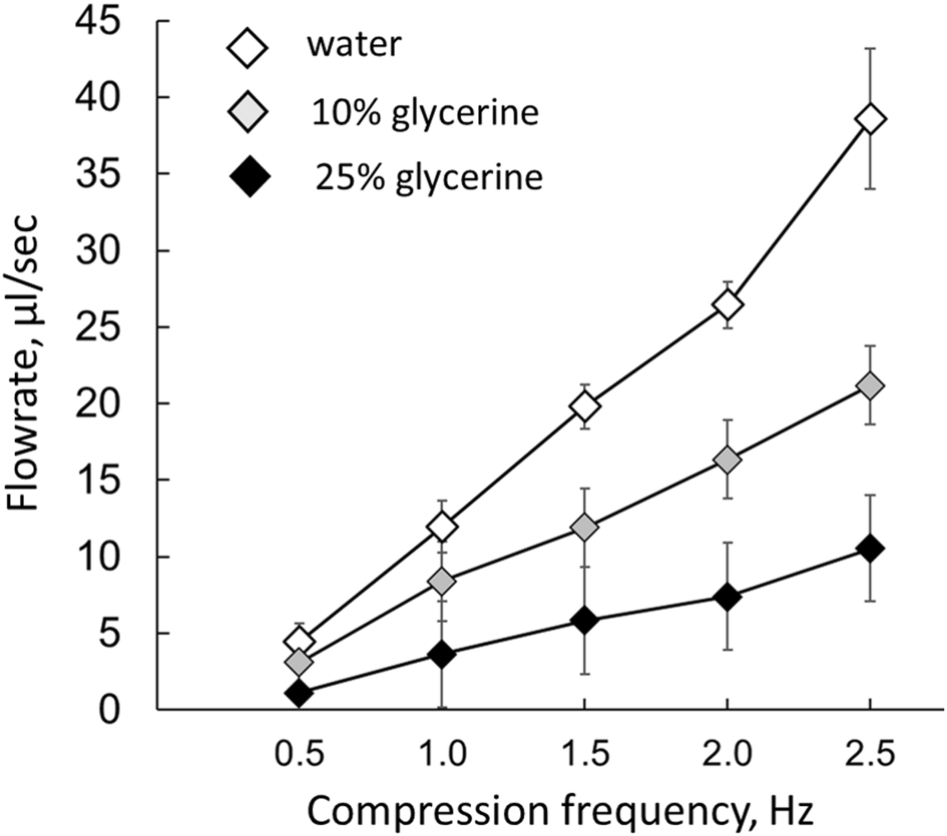
The flowrate–frequency relationship. Within the examined range of frequencies and viscosities, an increase in compression frequency led to a linear increase in the net flowrate. The values shown in this graph were acquired using a 38-mm-long, 5-mm-wide compliant segment with wall thickness of a 0.5 mm. The pincher was located 2 mm from the junction. The corresponding Womersley numbers are listed in the Appendix.

**Figure 4:**
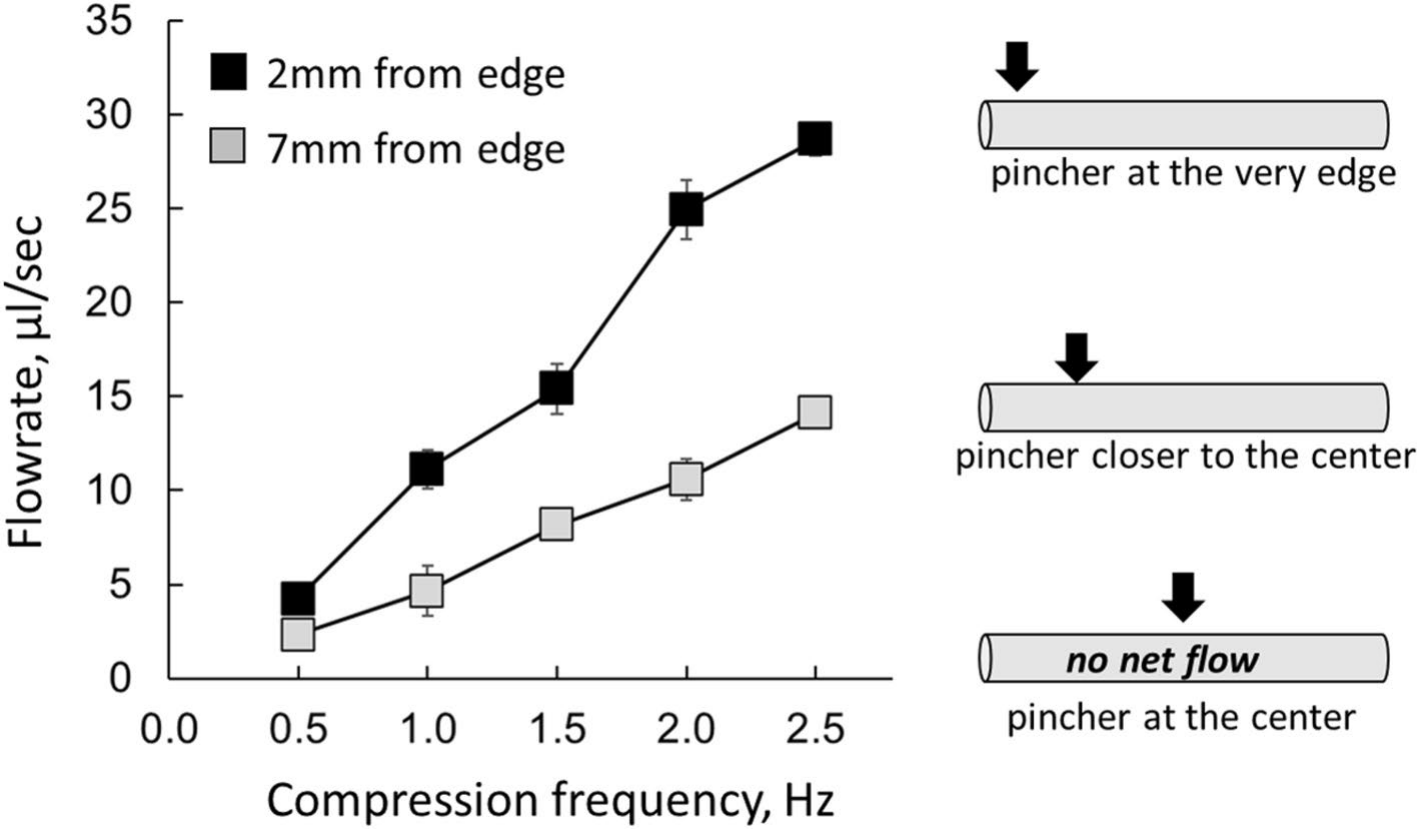
The flowrate–pincher position relationship. For all tested conduits, the maximal flow rates were observed when the pincher was at the very edge (i.e., the position closest to the junction between the compliant tube and the connector). No net flow was observed when pincher was located in the middle of the tube. The values shown in this graph were acquired using a 27-mm-long, 5-mm-wide compliant segment with a wall thickness of 0.4 mm with the loop filled with aqueous solution. The data shown are for the two pincher positions—2 mm and 7 mm from the junction between the stiff and compliant segments.

**Figure 5:**
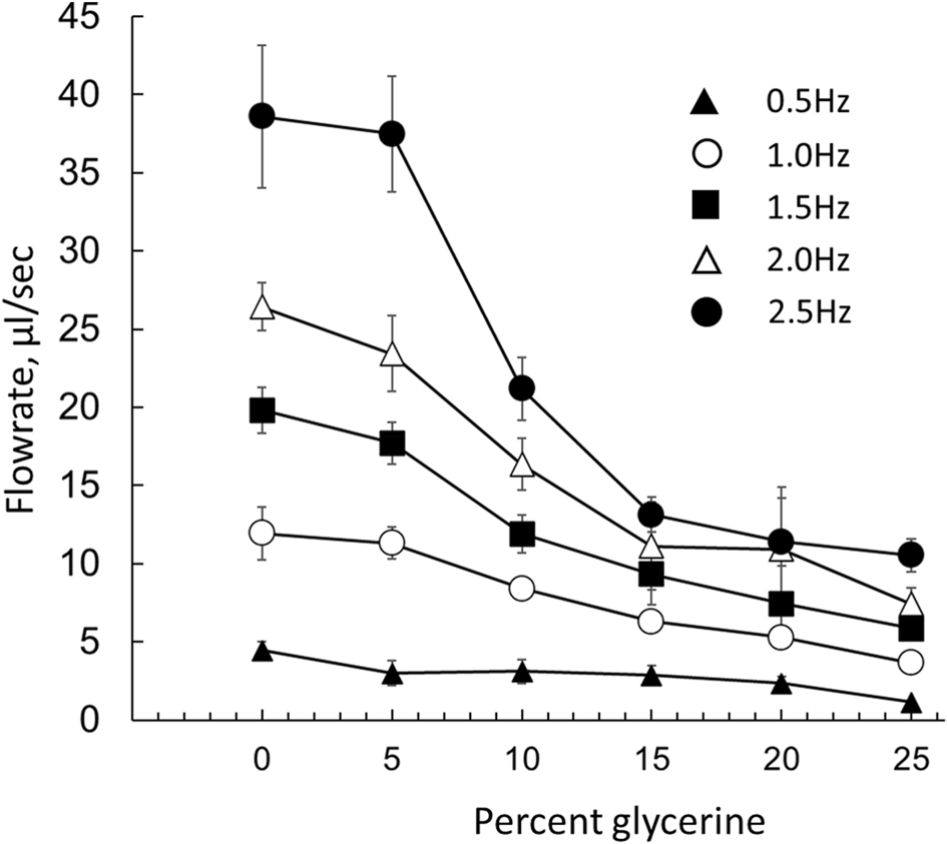
The flowrate–viscosity relationship. For the exception of 2.5 Hz, at all tested compression frequencies, increase in viscosity led to a linear decrease in net flowrates. The values shown were acquired using 38-mm-long, 5-mm-wide compliant segment with a wall thickness of 0.5 mm. The pincher was located 2 mm from the junction. The corresponding Womersley numbers are listed in the Appendix.

The velocity of the moving particles was derived from 60 frame/s video recordings taken from the transparent section of the Tygon tubing. To trace moving particles, a custom MATLAB particle tracking code was developed based on Jean-Yves Tinevez’s code using gap-closing and nearest-neighbors’ algorithms^21^. The mean velocity values were calculated from three separate 30-s long videoclips taken during each tested set of parameters. For the examined frequencies and vessel sizes, the calculated Womersley numbers were in the ~ 2–8 range based on formula W_o_ = r(ρω/µ)^1/2^, where r is the vessel radius, ω is the angular frequency, ρ is the fluid density, and µ is the dynamic viscosity. These Womersley numbers correspond to the intermediate regimes between the flat velocity profile characteristic for W_o_ > 10 and parabolic velocity profiles typical for W_o_ < 1. To calculate the mean flowrates for these intermediate regimes we used the Womersley number-based formula: Q = k_w_*A* v, where A stands for cross-sectional area of the Tygon tube and v is a time-averaged velocity of carbon particles flowing through mid-section of that tube. The Appendix includes Womersley numbers for the compliant tube segments used to produce Figs.  3, 4 and 5 data and their corresponding k_w_ values. The latter were calculated as detailed in Ponzini et al^22^.

## Results

### Making highly compliant conduits

Our first task was to create small cylindrical conduits with an elastic modulus comparable to what was reported for soft tissues, including heart muscle, i.e., a Young modulus in the range of 20–100 kP^23,24^. Commercially available medical tubing exceeds this range by at least two orders of magnitude. For example, tubing from Nordson MEDICAL, one of the largest medical device manufacturers, starts from durometer values of Shore A80, which corresponds to a Young modulus of ~ 10 MPa. Therefore, we sought to develop a protocol that could enable creation of vessels with biologically relevant dimensions and compliance values. Different materials and methods to create highly compliant conduits were tested, including the casting of various types of biocompatible polymers into alginate molds, the slow polymerization of tubes made from silicone rubber using a rotating platform, and extrusion-based techniques. Most of these attempts led to conduits that were either too fragile, too thick, or too heterogenous. The final and the most efficient, yet versatile and straightforward, approach is described in the  “Methods” section. It allowed us to create an arrangement of 1–5 cm long cylindrical tubes made of a thin, homogenous layer of highly compliant silicone rubber. The wall thickness of these conduits can be adjusted by layering a specific amount of the polymer mixture, while their inner diameter can be altered by using mandrels of different sizes. Tubes used in our experiments ranged from 0.3 to 1 mm and 3 to 8 mm in wall thickness and inner diameter, respectively. The standard deviation of the mean thickness value for the individual tubes was in the order of ~ 10%.

Notably, due to its slow polymerization rate, the silicone rubber is not a suitable material for 3D printing. However, if one is to make highly compliant tubular segments from biologically compatible ingredients or to create multilayered structures, 3D printing is definitely the method to be considered^25,26^.

### Flowrate versus compression frequency

When the pincher was positioned asymmetrically, all tested vessel sizes and compression frequencies yielded measurable flowrates with the movement of particles visible to the naked eye. Particles moved intermittently, in time with each compression event. This was particularly clear during the lowest compression frequencies of 0.5 and 1 Hz when the flow is in phase with created pressure gradients in accordance with the estimated Womersley numbers (See Appendix). An increase in compression frequency led to a linear rise in the mean velocity of the particles, with the highest compression frequencies yielding the highest mean flowrate. To ensure that measurements are made during the steady state regime at each individual frequency, recordings were taken 1 min after starting the actuator. The average values from three independent experiments were used to compile the graphs such as one shown in Fig. 3. For the size of compliant tube used in these experiments the maximal recorded flow velocity for the compression frequencies of 2.5 Hz reached 8.17 ± 0.96 mm/s which yielded a maximal flowrate of 38.6 ± 4.6   µl/sec or 2.3±0.3  ml/min.

### Impact of the pincher position

The mean direction of particle movement was generally in accordance with what was initially described by Liebau, which was from the pincher area toward the nearest junction point between the soft and stiff tubing: in other words, the fluid went from the longer portion of the elastic tube toward the shorter portion. When the pincher was positioned in the middle, the particles shifted back and forth during each compression event but there was no mean flow. The closer the pincher was positioned to the junction between the compliant tube and the connector, the larger mean flow was recorded (Fig. 4). The degree of lumen compression had direct correlation with the net flow rate, with the maximum flow observed when the lumen was fully compressed.

### Effect of viscosity

The fluid viscosity was varied by inclusion of the glycerin. Tested solutions included water and 5%, 10%, 15%, 20% and 25% glycerin which at 22 °C corresponded to 0.95, 1.10, 1.31, 1.56, 1.87, and 2.26 centipoise, respectively. The latter values were calculated using an online viscosity calculator^27^. The mean flowrates decreased in a linear fashion with respect to the increase of fluid viscosity when dimensionless Womersley numbers were below ~ 5. Such a linear relationship is expected based on the Poiseuille law, which describes vessel resistance (R ~ µ*L/r^4^). Deviation from a linear relationship started to occur when the Womersley number started to increase, which happened at the highest tested frequency of 2.5 Hz using water as the fluid (Fig. 5).

## Discussion

The circulation of biological fluids is accomplished by multiple mechanisms. One of the most obvious examples is blood pumping by an actively contracting heart equipped with one-way valves. Other common cases of valve-assisted flow include venous circulation and the movement of lymph. Yet, during embryonic development of the mammalian hearts and in several adult species of invertebrate animals, circulation is known to exists in absence of developed valves^28,29^. The mechanisms of such valveless circulation remain to be fully understood. One of possible mechanisms is based on the Liebau principle^1^. In this paper we examined the simplest experimental configuration of the Liebau pump to show its basic biological feasibility. A number of theoretical and preliminary experimental studies have suggested that this basic configuration can be further modified to increase the mean flowrate. Such modifications include the insertion of kinks and bends^16^, the addition of cavities^30^, the duplication of compression points^31,32^, or the inclusion of an inner gelatinous layer^33^. All of these modifications mimic possible physiological scenarios and may further increase the efficiency of Liebau-based pumping.

The physics of Liebau-type pumping is surprisingly complex since there are multiple variables involved, each having potential impact on the direction and the amplitude of the flow^6,7,9,15,34^. When recorded over broad range of frequencies, the relationship between the compression frequency and the mean flowrate has been shown to be highly non-linear^11,17,18,35^. Yet, the overall shape of these non-linear relationships varies wildly across the above cited experimental studies. Furthermore, when it comes to the quantitative analysis of existing experimental data, one of the most controversial concepts is the role of the resonant or natural frequency (F_n_). It was spurred by an earlier study by Hickerson et al^17^ in which the authors argued that the peak flow occurs at multiples of F_n_. The latter was calculated based on the formula F_n_ = c/2L, where the c is the velocity of the pressure wave and the L is the length of the compliant segment. Unfortunately, the authors did not elaborate as to why the value of c = 0.59 m/s used in their calculations is more than ten times smaller than the value that can be calculated using the Moens–Korteweg equation. To add to the confusion, a follow-up paper by the same authors^36^, mentions a value of c being two orders of magnitude higher (c = 60 m/s). In which case, the actual F_n_ value would been much higher than the estimated values of 8.8 Hz reported by the authors.

Despite these seemingly confusing computations, the study by Hickerson et al^17^ provided many important insights since it was the first detailed experimental study of the Liebau pump mechanism. Therefore, conclusions from this paper led many to try tying flowrate peaks to F_n_ values calculated based on the formula F_n_ = c/2L. Yet most of these studies failed to confirm such a relationship, either because such peaks were observed well below F_n_ or because F_n_ was beyond experimentally reachable values. Several explanations as to why significant flow can be observed at frequencies much less than F_n_ have thus been suggested. Among them is the damping of F_n_ by friction and inertia forces or the need to include the remaining parts of the loop into the F_n_ calculation^35^. Although these factors can indeed decrease the F_n_, they can hardly explain how sizable flow can be observed at frequencies lower than F_n_ by the orders of magnitude. Another doubt regarding the role of F_n_ was casted by experimental^35^ and theoretical studies of Liebau pump^6,10^ which showed that at F_n_ the system finds itself in a resonance mode yielding standing waves of pressure within the vessel. The negative and positive flowrates are then observed on both sides of the F_n_, with flowrates passing zero point at the F_n_ value.

The F_n_ concept is based on the superposition of reflected pressure waves that can lead to resonance and lead to changes in flow direction. Yet, these phenomena can happen only when vessels are long enough for multiple pressure waves to fit within the length of the compliant segment. The above is unlikely to occur for conduits that are on a millimeter to centimeter scale, particularly at physiological frequencies. Let’s consider, for example, one of the tubes tested in our studies for which pressure wave velocity can be estimated using the Moens–Korteweg equation. Based on a Young modulus of 47kPA and the values of 5 mm and 0.4 mm for the diameter and wall thickness of the compliant segment, pressure wave velocity will be around 2 m/sec. Based on the formula F_n_ = c/2L, the F_n_ for such vessel will then be ~ 25 Hz. Such a frequency is clearly above the range of compression frequencies that both types of cross-striated muscle can create on a continuous basis. Smaller vessel dimensions, such as the ones expected in embryos, will further increase pressure wave velocity and the corresponding estimates of F_n_ values. Therefore, alternative mechanisms as to how flow is created should be reconsidered for a Liebau-based pump that functions at physiological scales. Such mechanisms have indeed been suggested by Liebau^1,2^ and others^15^. The latter study for example argued that the flow occurs toward the less compliant shorter segment of the elastic tube because the pumping region tends to exchange fluid with the longer and therefore the more compliant segment.

Notably, we were interested in the frequencies that can be created by compression devices built using mammalian,—more specifically human muscle cells. Published data suggest that the non-linearity of the Liebau pump is mainly observed around resonant peaks. As argued above, for medium and small sized vessels, the natural frequency values lie outside biologically feasible ranges, therefore non-linearity in the frequency–flowrate relationship is unlikely to be observed.

Importantly, our experiments confirm that the system does not need to be close to the value of F_n_ to generate sizeable flow. By the term “sizeable”, we mean flowrate values that are on the same scale as the ones expected from a peristaltic pump of the same dimensions^13,15^. At its maximum efficiency, such a peristaltic pump is projected to move the volume of fluid equal to the volume displaced by the pincher while an active compression wave passes through the entire length of the tube. The flowrate of such a putative peristaltic pump can be estimated by the following formula: Q = F^*^A^*^P, where P is the length of compression segment (i.e., pincher), A is the cross-sectional area of the lumen, and F is the compression frequency. For the maximum tested frequency of 2.5 Hz, using a pincher of 3.5-mm-width and a tube with an inner diameter of 5-mm, the estimated peristaltic flowrate yields  172 µl/s flowrate. Therefore, the measured flowrate of ~ 40 µl/s created by the Liebau-pump using the same setup, is about a quarter of the maximal possible flowrate created by the peristaltic pump of similar dimensions and compression frequency. Importantly though, this flowrate is achieved by active compression of less than 10% of the tube length, while in the case of a peristaltic pump the entire vessel is required to be engaged in the act of active compression.

Another useful set of data from our experiments are the flowrate–viscosity relationships shown in Fig. 5. Numerical studies have suggested that at high frequencies the increased viscosity of the fluid has two effects on the Liebau pump performance^37^: a decrease in the amplitude of the flow pulsations and a drop in the mean flowrates. Published confirmation of these theoretical predictions is lacking, with the exception of one experimental study that compared the performance of the Liebau pump using water versus a 1:1 water–corn syrup mixture^16^. Our experiments tested several compression frequencies using multiple solutions with different viscosity values, with the highest numbers approaching the viscosity of the blood. The data suggest that at low frequencies with Womersley numbers ranging from 1 to 10, the viscosity affects flowrate in a nearly linear fashion, most likely due to a proportional increase in the vessel resistance according to the Poiseuille law. The impact of viscosity in our experiments was more significant compared to the study that used a corn syrup mixture^16^. Their data suggests that a tenfold increase in viscosity leads to ~ three–fourfold decrease in net flow. In our system the same degree of change in net flow occurred when the viscosity values only doubled. The much smaller linear dimensions of our setup (a few cm vs 35 cm) may play a role behind these differences.

Lastly, one may wonder about the possible impact of non-Newtonian behavior of the blood on Liebau pump performance. To the best of our knowledge, there are no experimental or modelling studies addressing this question. One of the reasons is the technical difficulty associated with using blood instead of a simple aqueous solution. Another reason is the relatively large dimensions of tubing typically used to test the Liebau phenomenon. Blood exerts its non-Newtonian behavior mainly at the level of the arterioles and capillaries, i.e., vessels with a diameter on the order of 10 to 100 microns^38^. When it comes to medium-sized vessels, non-Newtonian behavior of the blood is believed to play a role mainly at the sites where vessels are branching, bending or suddenly narrowing^39^. Otherwise, in large- to medium-sized straight vessels the impact imposed by the non-Newtonian behavior on blood flow is considered negligible. Previous studies of the Liebau pump, including our experiments, used tubing with diameter on the order of millimeters to centimeters, which is much larger than that of arterioles or capillaries. In addition, these studies were performed using straight or slightly curved tube segments. As a result, little attention was given to the possible effects of non-Newtonian behavior on performance of the Liebau pump. Nevertheless, this is an interesting subject to pursue further.

To conclude, herein we presented experimental data that confirmed the feasibility of a Liebau-type pumping at physiologically relevant scales. The assembled setup had all key parameters within the ranges expected to occur biologically, including the Young’s modulus and the linear dimensions of the compliant segment, the compression duration and frequency, and the fluid viscosity. These data warrant creation of similar pumping systems using excised vessels and/or tissue-engineered components^25,40–42^. These key components can be positioned outside the vessel of interest and can include a pair of low compliance cuffs and a band of periodically contracting muscle. The later can be made from induced pluripotent stem cell (iPS) derived cardiomyocytes or a stimulable ring of skeletal muscle cells. The most attractive aspect of such a design is that the integrity of the inner endothelial layer does not have to be disrupted, avoiding possible fibrosis, blockage, or, in the case of blood flow, thrombi formation. Such biomimetic pumps can serve as energy-efficient flow generators in microdevices or can be used to model the function of embryonic hearts during normal development or in diseased states.

## Supplementary Information

Supplementary Information.

## Data Availability

All data analyzed for this study are included in this published article and its Supplementary Information file.

## References

[1] Liebau G (1955). Die Strömungsprinzipien des Herzens. Z. Kreislaufforsch..

[2] Liebau G (1954). Uber ein ventilloses Pumpprinzip. Naturwissenschaften.

[3] Randall DJ, Davie PS, Bourne GH (1980). The heart of urochordates and cephalochodates. Heart and Heart-like Organs.

[4] Johansen K, Burggren W, Bourne GH, Bourne GH (1980). Cardiovascular function in the lower vertebrates. Hearts and heart-like organs.

[5] Forouhar AS, Liebling M, Hickerson A, Nasiraei-Moghaddam A, Tsai HJ, Hove JR, Fraser SE, Dickinson ME, Gharib M (2006). The embryonic vertebrate heart tube is a dynamic suction pump. Science.

[6] Borzì A, Propst G (2003). Numerical investigation of the Liebau phenomenon. Z. Angew. Math. Phys..

[7] Auerbach D, Moehring W, Moser M (2004). An analytic approach to the Liebau problem of valveless pumping. Cardiovasc. Eng..

[8] Kenner T, Moser M, Tanev I, Ono K (2000). The Liebau-effect or on the optimal use of energy for the circulation of blood. Scr. Med. (Brno).

[9] Jung E (2007). A mathematical model of valveless pumping: A lumped model with time-dependent compliance, resistance, and inertia. Bull. Math. Biol..

[10] Takagi S, Takahashi K (1985). Study of a piston pump without valves: Pumping effect and resonance in a pipe-capacity-system with a t-junction. Bull. Jpn. Soc. Mech. Eng..

[11] Ottesen JT (2003). Valveless pumping in a fluid-filled closed elastic tube-system: One-dimensional theory with experimental validation. J. Math. Biol..

[12] Jung E, Peskin CS (2002). Two-dimensional simulations of valveless pumping using the immersed boundary method. SIAM J. Sci. Comput..

[13] Thomann H (1978). A simple pumping mechanism in a valveless tube. Z. Angew. Math. Phys..

[14] Wen CY, Chang HT (2009). Design and characterization of valveless impedance pumps. J. Mech..

[15] Bringley TT, Chilress S, Vandenberghe N, Zhang J (2008). An experimental investigation and a simple model of a valveless pump. Phys. Fluids.

[16] Hiermeier F, Männer J (2017). Kinking and torsion can significantly improve the efficiency of valveless pumping in periodically compressed tubular conduits. Implications for understanding of the form–function relationship of embryonic heart tubes. J. Cardiovasc. Dev. Dis..

[17] Hickerson AI, Rinderknecht D, Gharib M (2005). Experimental study of the behavior of a valveless impedance pump. Exp. Fluids.

[18] Rinderknecht D, Hickerson AI, Gharib M (2005). A valveless micro impedance pump driven by electromagnetic actuation. J. Micromech. Microeng..

[19] Lee VCC, Chai CH, Law MC, Wee SK (2017). On the analysis of impedance-driven reverse flow dynamics. J. Eng. Sci. Technol..

[20] Muselimyan N, Al Jishi M, Asfour H, Swift L, Sarvazyan NA (2017). Anatomical and optical properties of atrial tissue: Search for a suitable animal model. Cardiovasc. Eng. Technol..

[21] github.com/tinevez/simpletracker.

[22] Ponzini R, Vergara C, Rizzo G, Veneziani A, Roghi A, Vanzulli A, Parodi O, Redaelli A (2010). Womersley number-based estimates of blood flow rate in Doppler analysis: In vivo validation by means of phase-contrast MRI. IEEE Trans. Biomed. Eng..

[23] Egorov V, Tsyuryupa S, Kanilo S, Kogit M, Sarvazyan A (2008). Soft tissue elastometer. Med. Eng. Phys..

[24] Ogneva IV, Lebedev DV, Shenkman BS (2010). Transversal stiffness and Young’s modulus of single fibers from rat soleus muscle probed by atomic force microscopy. Biophys. J..

[25] Koti P, Muselimyan N, Mirdamadi E, Asfour H, Sarvazyan NA (2019). Use of GelMA for 3D printing of cardiac myocytes and fibroblasts. J. 3D Print Med..

[26] Mirdamadi E, Muselimyan N, Koti P, Asfour H, Sarvazyan N (2019). Agarose slurry as a support medium for bioprinting and culturing freestanding cell-laden hydrogel constructs. 3D Print Addit. Manuf..

[27] Online viscosity calculator. met.reading.ac.uk/~sws04cdw/viscosity_calc.html.

[28] Männer J, Wessel A, Yelbuz TM (2010). How does the tubular embryonic heart work? Looking for the physical mechanism generating unidirectional blood flow in the valveless embryonic heart tube. Dev. Dyn..

[29] Anderson RH (1981). Hearts and heart-like organs. Volume 1. Comparative anatomy and development. J. Anat..

[30] Kozlovsky P, Rosenfeld M, Jaffa AJ, Elad D (2015). Dimensionless analysis of valveless pumping in a thick-wall elastic tube: Application to the tubular embryonic heart. J. Biomech..

[31] Lee VCC, Abakr YA, Woo KC (2013). Valveless pumping using a two-stage impedance pump. Front. Mech. Eng..

[32] Rosenfeld M, Avrahami I (2010). Net flow rate generation by a multi-pincher impedance pump. Comput. Fluids.

[33] Loumes L, Avrahami I, Gharib M (2008). Resonant pumping in a multilayer impedance pump. Phys. Fluids.

[34] Manopoulos C, Tsangaris S, Mathioulakis D (2020). Net flow generation in closed-loop valveless pumping. Proc. Inst. Mech. Eng. Part C J. Mech. Eng. Sci..

[35] Meier, J. A novel experimental study of a valveless impedance pump for applications at Lab-On-Chip, microfluidic, and biomedical device size scales. *PhD Thesis, Calif Inst Technol*. (2011).

[36] Hickerson AI, Gharib M (2006). On the resonance of a pliant tube as a mechanism for valveless pumping. J. Fluid Mech..

[37] Timmermann S, Ottesen JT (2009). Novel characteristics of valveless pumping. Phys. Fluids.

[38] Sochi, T. Non-Newtonian rheology in blood circulation. arXiv:13062067 (2013).

[39] Valencia A, Zarate A, Galvez M, Badilla L (2006). Non-Newtonian blood flow dynamics in a right internal carotid artery with a saccular aneurysm. Int. J. Numer. Methods Fluids.

[40] Sarvazyan NA (2014). Thinking outside the heart: Use of engineered cardiac tissue for treatment of chronic deep venous insufficiency. J. Cardiovasc. Pharmacol. Ther..

[41] Li Z, Seo Y, Aydin O, Elhebeary M, Kamm RD, Kong H, Taher Saif MA (2019). Biohybrid valveless pump-bot powered by engineered skeletal muscle. Proc. Natl. Acad. Sci. U. S. A..

[42] Azizgolshani, H. Tissue engineering active biological machines: Bio-inspired design, directed self-assembly, and characterization of muscular pumps simulating the embryonic heart. *PhD Thesis, Calif Inst Technol*. (2013).

