# Supplementary material for: Output of a valveless Liebau pump with biologically relevant vessel properties and compression frequencies
Source: Sci Rep. 2021 Jun 1;11:11505. doi: 10.1038/s41598-021-90820-4 (PMC8169938; doi:10.1038/s41598-021-90820-4)
Supplement: Supplementary file 1 — Supplementary Information. [file 41598_2021_90820_MOESM1_ESM.pdf]

SUPPLEMENTARY INFORMATION FILE

for the manuscript
Output of a valveless Liebau pump with biologically relevant vessel properties and compression frequencies

Rubina Davtyan & Narine A. Sarvazyan

CALCULATION OF WOMERSLEY NUMBER

The following formula was used to calculated dimensionless Womersley number for each condition:

$$W_o = R * (\omega * \rho / \mu)^{1/2}$$

where R is the inner radius of the tube, ω is the angular frequency equal to 2πF, ρ is the fluid density, and μ is the fluid dynamic viscosity. The inner radius of the transparent segment of the Tygon tubing where particle tracking took place was 1.5mm. Values for density and dynamic viscosity of aqueous glycerin solutions at 22°C were taken from *http://www.met.reading.ac.uk/~sws04cdw/viscosity_calc.html.*
Dimensionless Womersley numbers are shown using blue font

**Womersley numbers, Wo**							
glycerin	***ρ, density***	***μ, dynamic viscosity***	***F, compression frequency (Hz)***				
%	***kg/m³***	***Ns/m²***	***0.5***	***1***	***1.5***	***2***	***2.5***
0	***997.61***	***0.0010***	2.71	3.84	4.70	5.43	6.07
5	***1012.20***	***0.0011***	2.53	3.58	4.38	5.06	5.66
10	***1026.90***	***0.0013***	2.35	3.32	4.07	4.70	5.26
15	***1041.50***	***0.0016***	2.17	3.07	3.76	4.34	4.85
20	***1056.20***	***0.0019***	1.99	2.82	3.45	3.99	4.46
25	***1070.7***	***0.0023***	1.82	2.57	3.15	3.64	4.07

WOMERSLEY NUMBER-ADJUSTED CALCUATION OF THE FLOWRATE

To calculate the mean flowrate Q the following formula was used: $Q = k_w * A * v$, where A is the cross-sectional of the transparent segment of the Tygon tubing where particle tracking took place and v is the linear velocity of the particles flowing through its mid-section. ***Correction coefficients k_w*** were calculated based on equations given by Ponzini, R et al (IEEE Transactions on biomedical engineering 57.7 (2010): 1807-1815) and are given below using green font:

**Correction coefficients, k_w**					
glycerin %	***F, compression frequency (Hz)***				
	0.5	***1***	***1.5***	***2***	***2.5***
0	0.540	0.625	0.647	0.660	0.669
5	0.532	0.617	0.640	0.654	0.663
10	0.526	0.607	0.632	0.647	0.657
15	0.521	0.597	0.623	0.639	0.650
20	0.516	0.567	0.612	0.630	0.641
25	0.512	0.534	0.600	0.619	0.632
